# Selective retinoid X receptor agonism promotes functional recovery and myelin repair in experimental autoimmune encephalomyelitis

**DOI:** 10.1186/s40478-024-01904-x

**Published:** 2024-12-21

**Authors:** Gracious D. S. Kasheke, Basmah A. M. Hendy, Gabriel G. Dorighello, Nonthué A. Uccelli, Jean-David M. Gothié, Robyn J. Novorolsky, Madison J. Oulton, Jude Asainayagam, Adam I. Makarov, Kaitlyn S. Fraser, Vidyasagar Vuligonda, Martin E. Sanders, Timothy E. Kennedy, George S. Robertson

**Affiliations:** 1 Department of Pharmacology, Faculty of Medicine, Dalhousie University, Halifax, NS B3H 4R2 Canada; 2 Brain Repair Centre, Life Sciences Research Institute, Dalhousie University, Halifax, NS B3H 4R2 Canada; 3 Department of Psychology and Neuroscience, Faculty of Science, Dalhousie University, Halifax, NS B3H 4R2 Canada; 4 Department of Neurology and Neurosurgery, Montreal Neurological Institute, McGill University, Montreal, QC H3A 2B4 Canada; 5 Io Therapeutics, Inc, Spring, TX 77387 USA; 6 Department of Psychiatry, Faculty of Medicine, Dalhousie University, Halifax, NS B3H 2E2 Canada; 7 1348 Summer St, Halifax, NS B3H 0A8 Canada

**Keywords:** Multiple sclerosis, Neural repair, Neuroprotection, Oligodendrocyte progenitor cell, Rehabilitation

## Abstract

Evidence that myelin repair is crucial for functional recovery in multiple sclerosis (MS) led to the identification of bexarotene (BXT). This clinically promising remyelinating agent activates multiple nuclear hormone receptor subtypes implicated in myelin repair. However, BXT produces unacceptable hyperlipidemia. In contrast, IRX4204 selectively activates the retinoid X receptor (RXR). Given compelling links between RXR activation and increased myelin repair, we employed IRX4204 to investigate the impact of RXR agonism alone on functional recovery in mice subjected to experimental autoimmune encephalomyelitis (EAE). Since gait deficits are common in MS, we used machine learning to obtain highly sensitive and reliable measurements of sagittal hindleg joint movements for mice walking on a treadmill. IRX4204 not only blocked the progressive loss of knee and ankle movements but also reversed joint movement impairments in EAE mice. Our biochemical, transcriptional and histological measurements in spinal cord suggest these gait improvements reflect increased axon survival and remyelination and reduced inflammation. Using microglia, astrocytes and oligodendrocyte progenitor cells, we present additional data suggesting that IRX4204 may act on multiple glial subtypes to orchestrate myelin repair. These results inform the discovery of restorative neural therapeutics for MS by demonstrating that selective RXR agonism is sufficient for effective myelin repair. Moreover, our findings support the therapeutic potential of IRX4204 to promote functional recovery in MS.

**Supplementary Information:**

The online version contains supplementary material available at 10.1186/s40478-024-01904-x.

## Introduction

MS is characterized by autoimmune-mediated demyelination in the central nervous system (CNS) that promotes axonal damage, ultimately leading to axonal loss that drives disease progression [8, 13, 30, 32, 57, 62, 66]. Remyelination is therefore considered crucial for functional recovery in MS [9, 37]. This has sparked an intense search for therapies that can enhance remyelination to slow, halt, or reverse MS disease progression. These studies identified bexarotene (BXT) as a putative remyelinating agent for MS [43]. A recent clinical trial found that BXT produced durable improvements in electrophysiological and imaging correlates of remyelination but could not be tolerated due to adverse side effects [10, 11, 40]. BXT activates several nuclear hormone receptors including RXR, retinoic acid receptor (RAR), liver X receptor (LXR), and peroxisome proliferator-activated receptor (PPAR) that form homodimers and heterodimers which activate complex transcriptional programs [53]. This broad activity on nuclear hormone receptors is thought to enable BXT to regulate the expression of a vast array of genes that enhance the proliferation of vascular and neural progenitor cells as well as the polarization of immune and glial cells to pro-repair phenotypes to resolve inflammation, clear cellular debris, and create a fertile environment essential for CNS repair [7, 17, 43, 48]. However, it may not be necessary to activate such a broad set of nuclear hormone receptors to promote myelin repair. Indeed, there is a well-established link between RXR activation and remyelination. RXR inhibition disrupts remyelination while 9-cis-retinoic acid, a vitamin A metabolite that activates RAR and RXR, accelerates remyelination in mice [26, 43]. These findings suggest that RXR agonism alone might oppose disease progression and restore neurological function in MS.

The neurorestorative properties of RXR activation led to the development of the preferential RXR agonist IRX4204. IRX4204 is orally bioavailable and 1000 times more potent at RXRs (α, β, and γ) than RARs (α, β, and γ) [50, 60]. Unlike the FDA-approved RXR agonist, BXT, IRX4204 may be less likely to cause adverse cardiovascular side effects because it has much greater selectivity for the RXR [35, 50, 60]. When administered early in the disease course, IRX4204 has been shown to resolve inflammation and reduce paralysis in mice subjected to EAE [16]. Hence, we used IRX4204 to determine whether RXR agonism is sufficient to promote functional recovery in female EAE mice when the onset of administration was delayed until peak disease.

Walking (gait) deficits are one of the most disabling features of MS that increase the risk of injurious falls [5, 14, 38]. Approximately 70% of MS patients suffer from gait deficits that can be observed in the absence of clinical disability [38, 42]. Impaired gait in MS is characterized by reduced range of motion (ROM) and increased variability in leg joint movements [15]. We have previously characterized gait deficits in mice subjected to EAE [18, 21, 22]. In these studies, kinematic gait analysis was performed to measure joint movements of a hind leg in the sagittal plane of mice while they walked on a treadmill. Kinematic gait analysis yielded highly sensitive and accurate measurements of walking deficits in EAE mice that resemble those seen in MS [18, 21, 22]. Furthermore, increased variability of knee and ankle joint movements was highly correlated with myelin loss in the spinal cord [22]. Moreover, we have shown that kinematic gait analysis is a useful behavioural tool to identify promising restorative drugs for MS [18].

The present study therefore used kinematic gait analysis to examine the effects of oral IRX4204 administration beginning at peak disease on functional recovery in EAE mice. These behavioural studies were complemented by transcriptional and histological measurements that assessed the effects of delayed IRX4204 administration on inflammation, remyelination, and axon integrity in the spinal cord. To better understand how preferential RXR activation impacts neural cells to resolve inflammation and stimulate remyelination, we examined the effects of IRX4204 on microglia, astrocytes, and oligodendrocyte progenitor cells (OPCs) that mediate myelin repair. In these studies, we examined the concentration dependent effects of IRX4204 on microglial and astrocytic polarization to a pro-inflammatory or pro-repair phenotype, and the differentiation of OPCs into myelin-producing oligodendrocytes.

## Methods

### Experimental animals

Studies on clinical scores, gait, white matter loss, and in vivo gene expression were all performed on adult female C57BL/6 mice aged 12 − 14 weeks at the beginning of the studies. Time-pregnant CD1 mice and Sprague Dawley rat pups were used to obtain primary astrocyte and primary OPC cultures, respectively. These animals were obtained from Charles River Canada (Charles River, MA, USA). Female mice expressing a neuronal-specific Thy1-eYFP construct on a C57BL/6 background were used to assess axonal injury by the quantification of punctate eYFP fluorescence (The Jackson Laboratory; Stock No: 012708, ME, USA). All studies were performed in accordance with the Canadian Council on Animal Care guidelines and were approved by the Dalhousie University Committee on Laboratory Animals or the Montreal Neurological Institute Animal Care Committee. Mice were housed in the Life Science Research Institute Animal Care Facility on a 12-hr light/dark cycle (7:00 am/7:00 pm); food and water were provided ad libitum. Mice were allowed one week to habituate to the facility prior to experimentation. At the end of all in vivo studies, mice were euthanized with an intraperitoneal (i.p.) injection of 200 µL of sodium pentobarbital (34 mg/mL; Schering Canada, QC, Canada) and intracardially perfused with PBS (10 mL; pH = 7.4).

### EAE induction

A peptide fragment corresponding to amino acid sequence 35–55 of mouse myelin oligodendrocyte glycoprotein (MOG_35–55_; MEVGWYRSPFSRVVHLYRNGK; Gen Script, NJ, USA) was dissolved in phosphate buffered saline (PBS; pH = 7.4) at 3 mg/mL. This solution was then emulsified in complete Freund’s adjuvant (CFA) at a 1:1 ratio by volume. To induce EAE, mice received bilateral 100 µL subcutaneous (s.c.) injections of this final mixture on day post-immunization (DPI) 00. MOG_35–55_ immunization controls received bilateral s.c. injections of PBS (pH = 7.4) emulsified in CFA at a 1:1 ratio. All mice also received a first 200 µL intraperitoneal (i.p.) injection of pertussis toxin (Sigma-Aldrich, MO, USA) dissolved in water at 1.5 µg/µL on DPI 00 and a second injection on DPI 02.

### Clinical scoring

Behavioural deficits associated with increasing disease severity were evaluated using the following ordinal clinical scoring scale: 0, no clinical signs; 0.5, hooked tail; 1, flaccid tail; 1.5, flaccid tail with splay; 2, minor walking deficits, mild ataxia; 2.5, severe walking deficits; 3, dropped pelvis in addition to severe walking deficits, chronic ataxia; 3.5, unilateral hindlimb paralysis; 4, bilateral hindlimb paralysis; 4.5, forelimb paralysis; 5, moribund. Two trained observers began clinical scoring at DPI 07 while blinded to the experimental conditions.

### Experimental groups and dosing

For the experimental group designated as EAE/Veh, mice were dosed with 5 mL/kg of vehicle (NEOBEE^®^ 1053; Stepan, Northfield, IL, USA). For the experimental group designated as EAE/IRX4204, mice were dosed with 12 mg/kg IRX4204 (Io Therapeutics Inc., TX, USA). All doses were administered once daily by oral gavage (p.o.). CFA (DPI 16) and EAE (DPI 16) mice from the in vivo gene expression studies were not dosed with vehicle nor with IRX4204 and were euthanized at peak disease (DPI 16) to act as controls for EAE induction.

### Kinematic gait analysis

**Fig. 1:**
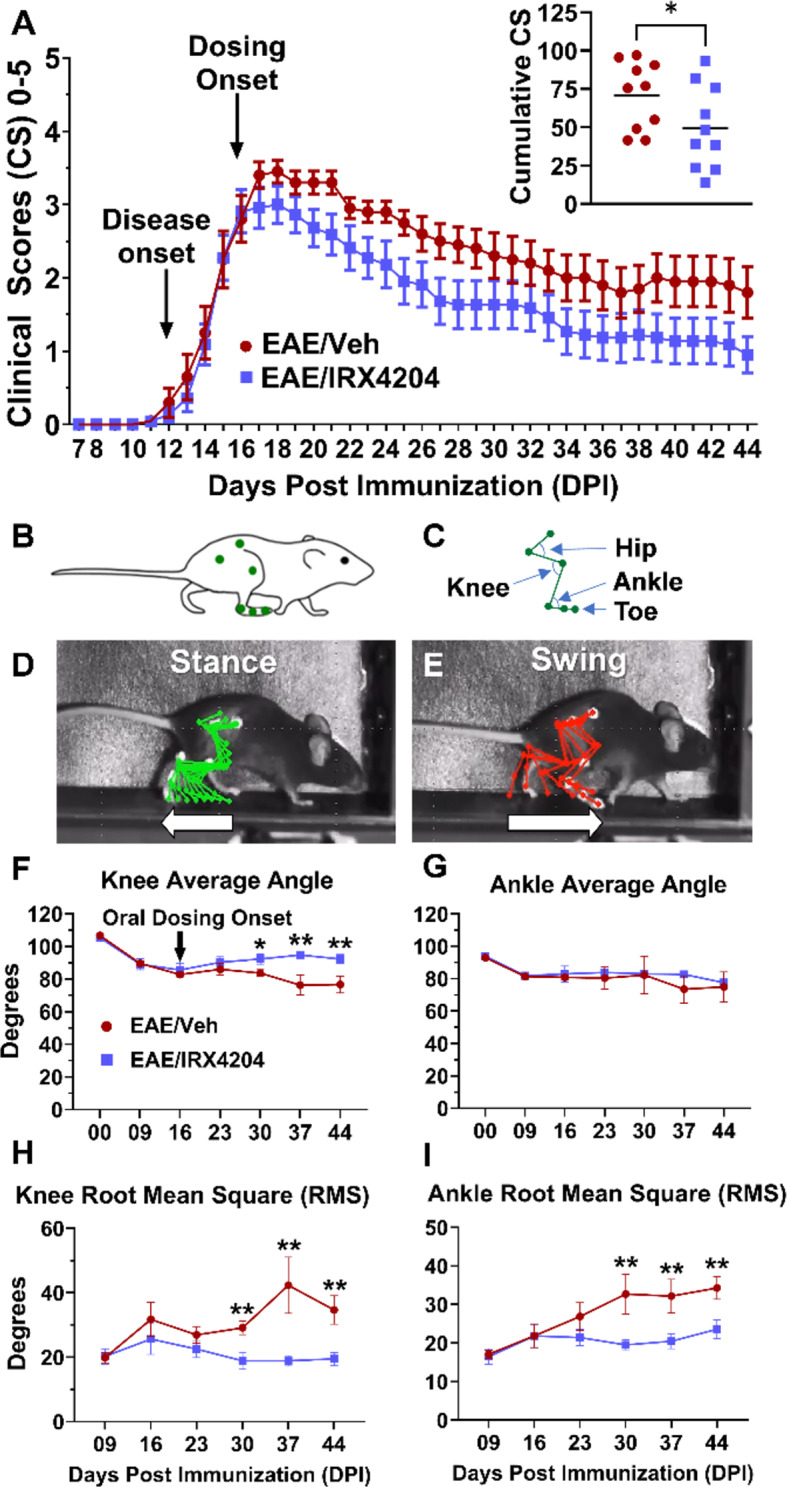
Oral administration of IRX4204 beginning at peak disease (DPI 16) reduces clinical scores and reverses gait deficits in EAE mice. (**A**) Daily clinical scores from DPI 07–44 for EAE mice treated with vehicle (Veh) or IRX4204 (mean ± SEM). Relative to Veh (5 mL/kg/day, po), IRX4204 (12 mg/kg/day, po) administration beginning at peak disease (DPI 16) reduced cumulative clinical scores (insert; *n* = 10, one-tailed Mann-Whitney *U* test, *P* < 0.05, mean ± SEM). (**B**–**E**) Six points of the hindlimb (**B**) were tracked to construct a stick model for leg movements (**C**) during the stance (**D**) and swing (**E**) phases of a gait cycle while mice walked on a treadmill. (**F**–**I**) Gait was measured at baseline (DPI − 02) and DPI 9, 16, 23, 30, 37 and 44. EAE resulted in gait deficits by DPI 16. Relative to Veh, IRX4204 partially reversed the loss of knee average angle (**F**), completely normalized knee RMS differences (**H**) and prevented the progressive increase of ankle RMS differences (**I**) from DPI 30–44 (*n* = 10, two-way ANOVA, Šidák’s multiple comparisons test, mean ± SEM; *p < 0.05, **p < 0.01)

Movements of the right hindlimb were recorded in the sagittal plane as mice walked on a treadmill. A baseline gait recording was taken for each mouse two days prior to the induction of EAE (DPI − 02). Subsequent recordings occurred at weekly timepoints beginning on DPI 09. To ensure that all the mice included were able to walk, kinematic gait analysis was only performed on mice with mild EAE symptoms (clinical score ≤ 2.5). Video recordings were performed using a high-speed camera at a frame rate of 250 frames/s. DeepLabCut software was trained to track the iliac crest, hip, knee, ankle, metatarsophalangeal joint, and toe of mice during each video frame (Fig. 1C and D). This software produced CSV files of X and Y pixel coordinates corresponding to the position of each joint on a frame-by-frame basis. For conversion of pixel distances to centimeter (cm) values, an additional calibration video containing four markers arranged in a 4 cm (height) by 7 cm (width) rectangle was recorded at each timepoint. This calibration recording was taken under the same videographic conditions as those used for all recordings taken at that respective timepoint. One frame from this calibration video was used as input for ImageJ which calculated the conversion coefficient for pixel values to cm values based on the indicated distances between markers. The conversion coefficient and CSV files were used as input into a customized R script. KinemaR used the conversion coefficient to transform the pixilated data into cm values allowing for measurements of joint height and the measurement of hip, knee, and ankle angle in each frame. Phase detection and data normalization were performed by KinemaR as previously described [2, 21, 22, 47]. Briefly, the swing and stance phases (Fig. 1E and F) were detected for each step cycle in a video. Swing and stance phases were each normalized to 100 frames. All of the normalized swing phases and stance phases within a video were then averaged to produce a single representative swing phase and a single representative stance phase of 100 frames each. Together, these comprised the 200-frame representative step cycle for each recording. The representative step cycle was used to calculate average angles for the knee and ankle joints. Joint angles for each frame of the normalized and averaged step cycle were used to calculate the root mean squared (RMS) difference.

### Tissue preparation for histology and fluorescence microscopy

EAE/Veh and EAE/IRX4204 mice were euthanized at DPI 44. Mice were then transcardially perfused with PBS followed by 4% paraformaldehyde (PFA; 10 mL; pH = 7.4; Thermo Fisher Scientific, MA, USA) immediately following perfusion with PBS. Spinal cords were dissected from the spinal column by laminectomy and post-fixed in 4% PFA for 24 h. The spinal cords were then transferred to 15% sucrose for 24 h followed by 30% sucrose for 24 h. Next, spinal cord segments L2 − 5 were dissected, embedded in Tissue-Tek^®^ optimal cutting temperature (OCT; Sakura^®^ Finetek, CA, USA) and frozen on dry ice. The embedded samples were then mounted into a Leica CM1950 cryostat set to -18 °C. Serial sagittal sections were cut at a thickness of 30 μm and mounted on Superfrost glass slides (Thermo Fisher Scientific, MA, USA).

### Eriochrome cyanine staining

Slides containing sagittal spinal cord sections were dipped into descending concentrations of ethanol at 100%, 90%, and 75% for rehydration. Sections were then rinsed in tap water at room temperature and immersed for 15 min in a 0.16% aqueous solution of Eriochrome Cyanine (EC; Sigma Aldrich, MO, USA) containing 0.4% H_2_SO_4_ and 0.4% FeCl_3_. Sections were then differentiated in 0.5% ammonium hydroxide and counterstained in a 1% solution of Neutral Red (NR; Acros Organics, NJ, USA) for 2 min. Sections were then dipped into a series of increasing concentrations of ethanol at 75%, 90%, and 100% for dehydration prior to being cleared with xylene. Slides were then cover-slipped using Cytoseal 60 (Thermo Fisher Scientific, MA, USA) for imaging using a Zeiss AxioStar Plus (Zeiss, Baden-Württemberg, Germany). Imaging parameters remained the same for all sections. EC stains white matter in blue following differentiation with ammonium hydroxide while NR counterstaining exposes areas where myelin is absent [56]. This dual staining technique was used to quantify white matter loss by measuring the total area of NR staining within white matter regions.

### Fluorescence microscopy

Slides containing sagittal spinal cord sections from Thy1-cre/ERT2-eYFP expressing mice were stored at -20 °C in a light-resistant slide box. Sections were brought to room temperature then imaged for endogenous eYFP fluorescence using a Zeiss Axio Imager Z2 with a monochrome camera (Zeiss, Baden-Württemberg, Germany). Bright, punctate labelling in white matter regions of the spinal cord, representative of eYFP aggregation, was used as a marker of axonal transection as described in earlier studies [25]. To discern distinctive areas of bright eYFP labelling in white matter, a threshold was applied to each image. Because the average pixel fluorescence intensity in grey matter was not affected by EAE or treatment groups, threshold values for each image were applied with respect to average grey matter fluorescence. Normalization to grey matter fluorescence provided a final threshold value for each image which accounted for stochastic variation in fluorescence between spinal cord sections. The threshold value was calculated for each image in several steps. First, average pixel fluorescence intensity in healthy appearing white matter was divided by average pixel fluorescence intensity in grey matter in each image. These values were then averaged to obtain a standardized ratio for healthy white matter fluorescence normalized to grey matter fluorescence. Finally, the standardized ratio was multiplied by the average pixel fluorescence intensity of grey matter in each image followed by a factor of 1.5 to obtain a threshold for each image. The percent area of white matter pixels with suprathreshold fluorescence was then calculated over the total white matter area as a measure of percent area axonal transection.

### Transmission electron microscopy

EAE/Veh and EAE/IRX4204 mice were euthanized at DPI 30 and intracardially perfused with PBS followed by 2.5% glutaraldehyde (Fisher Scientific, New Jersey, USA) diluted in 0.1 M Sodium Cacodylate Buffer (4 mL; pH = 7.4). Spinal cords were dissected from the spinal column by laminectomy and post-fixed in 2.5% glutaraldehyde for 24 h. Spinal cord segments L3 − 4 were then dissected, rinsed 3 times in 0.1 M sodium cacodylate buffer and fixed in 1% osmium tetroxide for 2 h and, after dehydration, embedded in epon araldite resin. Section 100 nm thick were cut with an ultramicrotome and placed on mesh copper grids, stained with 2% aqueous uranyl acetate, rinsed, and treated with lead citrate, then rinsed again and air dried. Images were captured with a Jeol Jem 1230 transmission electron microscope equipped with a Hamatsu ORCA-HR digital camera at 80 kV and 10 000× magnification. Image analysis for axonal counts and g-ratio calculations was performed using the AxonDeepSeg model model_seg_mouse_axon-myelin_tem [68]. Following automated image analysis, editing of the automated segmentation was performed by an experimenter blinded to experimental conditions to ensure high quality segmentation. Myelinated axons with g-ratios above 0.8 were considered remyelinating axons based on prior characterization in the EAE mouse model [41].

### C8-B4 microglial cultures

The C8-B4 microglia cell line (ATCC, VA, USA) was maintained in Dulbecco’s Modified Eagle Medium (DMEM; pH = 7.4; Thermo Fisher Scientific, Cat. # 11995065, MA, USA). For the nitrate release and phagocytosis assays, C8-B4 microglia were seeded in 48-well plates at a density of 150 000 cells/well. To measure gene expression, the cells were seeded in 24-wells plates at a density of 300 000 cells/well. In these assays, the C8-B4 microglial wells were pre-treated with either PBS (pH = 7.4) 0.1, 1, or 10 nM of IRX4204 for 24 h, prior to the addition of lipopolysaccharide (LPS; 100 ng/mL; Sigma-Aldrich, MO, USA) or PBS (pH = 7.4). All assays were performed 24 h following the addition of LPS or PBS (basal conditions).

### Microglial nitrate release assay

Cell media from C8-B4 microglial cultures was collected for measurement of nitrate levels using the Greiss reagent in a colorimetric assay, according to manufacturer instructions (Invitrogen™ - G7921, MA, USA). To account for differences in cell abundance between wells, the nitrate levels were normalized to the intensity of crystal violet DNA-staining as measured using the SPECTROstar Nano Absorbance Reader (BMG Labtech, Ortenberg, Germany).

### Microglial phagocytosis index

To assess microglial phagocytic activity, uptake of zymosan in these cells was measured in culture. This assay was performed as previously described in Dorighello et al. 2022 [19]. 10 µL of neutral-red stained zymosan (1 × 108 particles/mL) was added to each well and the cells were incubated in 5% (v/v) CO_2_ for 30 min. Next, the medium was removed, and the cells were fixed with Baker’s solution [4% (w/v) formaldehyde, 2% (w/v) sodium chloride, 1% (w/v) calcium acetate] at 37 °C for 30 min. The macrophages were washed twice with PBS and the neutral-red stain was solubilized with 0.1 mL of acidified alcohol solution [10% (v/v) acetic acid, 40% (v/v) ethanol in distilled water]. After 30 min, the absorbance was monitored at 550 nm using the SPECTROstar Nano Absorbance Reader (BMG Labtech, Ortenberg, Germany). The phagocytosis index was expressed related to crystal violet DNA staining in control wells for normalization to cell abundance.

### Astrocyte cultures

Astrocytes were isolated from cerebral cortices of CD1 pups (Charles River Canada, QC, Canada) at postnatal day 0 (P0) mice, as described in Novorolsky 2023 [46]. Following isolation, astrocytes were maintained in astrocyte media [DMEM high glucose (pH = 7.4; Life Technologies, CA, USA) supplemented with 10% FBS (HyClone Laboratories Inc., UT, USA) and 20 µg/mL gentamycin (Thermo Fisher Scientific, MA, USA)] until they reached confluency [day in vitro 7–9 (DIV 7–9)]. These cells were then passed and seeded in 12-well plates at a density of 100 000 cells/well. Plated wells were pre-treated with either PBS (pH = 7.4), 1, 10, or 100 nM of IRX4204 for 24 h prior to the addition of LPS (100 ng/mL; Sigma-Aldrich, MO, USA) or PBS (pH = 7.4). All assays were performed 16 h following the addition of LPS or PBS (basal conditions).

### OPC cultures

OPCs were isolated by shake-off from a mixed glial culture derived from the cerebral cortices of P2 rat pups, as previously described [3, 27, 39]. These cells were kept in SATO medium (DMEM supplemented with 5 µg/mL insulin, 50 µg/mL transferrin, 30 nM sodium selenite, 6.3 ng/mL progesterone, 16 µg/mL putrescine, 100 µg/mL penicillin/streptomycin, and 2 mM glutamax) without thyroxine or triiodothyronine at 37 °C in 5% CO2 for 1 day. Finally, OPCs were treated with 1:50,000 DMSO (negative control) or IRX4204 at 0.1, 1, 10 and 100 nM for 1 or 5 days.

### OPC Immunocytochemistry

After 2 or 6 DIV cells were fixed for 10 min with 4% PFA in PBS, washed in PBS, and blocked with 2.5% bovine serum albumin and 2.5% horse serum with 0.05% Triton X-100 in PBS for 30 min at room temperature. Cells were incubated overnight at 4 °C with a primary antibody against MBP (1:1000; Aves, #MBP0020) in blocking solution. Upon primary antibody washes, cells were fluorescently labeled with the secondary antibody (1:1000; Alexa Fluor 488, Invitrogen™, MA, USA) diluted in PBS with 2.5% bovine serum albumin and 2.5% horse serum and incubated for 1 h at room temperature. Nuclei were stained with 5 µg/mL Hoechst dye (Sigma-Aldrich, MO, USA) together with the secondary antibody incubation, and three washes with PBS were performed before mounting the coverslips with Dako mounting media. Four randomly chosen areas of each coverslip (4 replicates per condition) were imaged with a 20× objective using a Zeiss Axio Observer.Z1 microscope with an Axiocam 506 camera. Counting MBP positive cells was done manually using the cell counter plugin in ImageJ.

### Reverse transcription quantitative polymerase chain reaction (RT-qPCR)

CFA and EAE mice were euthanized at DPI 16 followed by EAE/Veh and EAE/IRX4204 mice at DPI 23 by an overdose injection of pentobarbital. Mice were then intracardially perfused with PBS (10 mL; pH = 7.4). Hydraulic extrusion of the spinal cord was performed using a blunted 18-gauge needle attached to a 10 mL syringe filled with PBS. Spinal cords were then flash-frozen using liquid nitrogen and stored at -80 °C until homogenization in 800 µL pureZOL™ RNA isolation reagent (Bio-Rad Laboratories, Inc., CA, USA) using a bead homogenizer (Benchmark Scientific, NJ, USA). Total RNA was extracted using the Aurum Total RNA Fatty and Fibrous Tissue kit (Bio-Rad Laboratories, Inc., Cat. #732–6870, CA, USA) following the spin protocol as per manufacturer instructions. For C8-B4 microglia and primary astrocyte cultures, total RNA was extracted using the Aurum total RNA minikit (Bio-Rad Laboratories, Inc., Cat. # 732–6820, CA, USA) following the spin protocol as per manufacturer instructions. Concentration and purity of the RNA was estimated spectrophotometrically upon elution using a SPECTROstar Nano spectrophotometer (BMG Labtech, Mandel Scientific Company Inc., ON, Canada). Quality and overall integrity of the isolated total RNA was measured on the Experion™ Automated Electrophoresis System (Bio-Rad Laboratories, Inc., CA, USA) using the Experion RNA StdSens Starter Kit (Bio-Rad Laboratories, Inc., CA, USA). Only samples with RNA Integrity Number (RIN) values of 7.5 or more were considered acceptable and used for further analysis. Generation of complementary DNA (cDNA) was performed according to manufacturer instructions with iScript™ cDNA Synthesis Kit (Bio-Rad Laboratories, Inc., CA, USA) using 850 ng of template from each tissue sample, 600 ng of template from each microglial well, and 180 ng of template from each astrocyte well. cDNA was stored at -20 °C. All qPCR experiments were performed in accordance with the MIQE guidelines on the Bio-Rad CFX96 Real-Time System C1000 Touch Thermal Cycler (Bio-Rad Laboratories, Inc., CA, USA) using the SsoFast EvaGreen Supermix Kit (Bio-Rad Laboratories, Inc., CA, USA) [12]. Each individual gene was optimized for annealing temperature. The RT-qPCR protocol for melt curve analysis used the following conditions: (95 °C for 30 s) + (95 °C x 5 s + 60 °C x 5 s + fluorescence read) x 40 cycles + melt curve analysis. The melting curve program used a 2 s hold time with plate readings for every 0.5 °C increase from 65 °C to 95 °C. ^ΔΔ^Cq method was used for data analysis using CFX Maestro software (Bio-Rad Laboratories, Inc., CA, USA). HPRT1 and GAPDH were selected as reference genes for the animal and astrocyte studies while GAPDH and β2M were selected as reference genes for the microglial culture studies based on geNorm *M* values below one for tissue samples and 0.5 for cell cultures. Statistical comparisons were performed using the average value of triplicate technical replicates for all experiments. Primer sequences can be found in supplementary materials.

### Statistical analyses

Cumulative clinical scores were calculated for each mouse by adding the daily clinical scores from DPI 16 − 44. For EAE studies, a one-tailed Mann-Whitney *U* test was used to analyze cumulative clinical scores, percentage of white matter loss, percentage of axonal transection, gene expression in vivo, number of remyelinating axons/µm^2^, and percentage of remyelinating axons. A two-way repeated measures ANOVA followed by Sidak’s multiple comparisons test was used to analyze gait data at DPI 16, 23, 30, 37, and 44. An ordinary one-way ANOVA followed by Dunnett’s multiple comparisons test was used to analyze the effect of increasing concentrations of IRX4204 on gene expression in vitro, microglial nitrate release, and microglial phagocytosis experiments. A non-parametric Kruskal-Wallis test followed by Dunn’s multiple comparisons test was used to analyze the effect of increasing concentrations of IRX4204 on the proportion of MBP-positive cells to DAPI-positive cells and the effect of increasing concentrations of IRX4204 on the number of DAPI-positive cells per mm^2^ in OPC cultures. All statistical analyses were performed using GraphPad Prism 8.0.

## Results

### Oral treatment with IRX4204 beginning at peak disease enhances recovery from EAE motor deficits

To assess the ability of IRX4204 to promote recovery in EAE mice, we administered either vehicle (5 mL/kg/day, p.o.) or IRX4204 (12 mg/kg/day, p.o.) beginning at peak disease (DPI 16). From DPI − 02–16, clinical scores remained similar between both groups (Fig. 1A). EAE mice treated with IRX4204 showed improved recovery after peak disease compared to EAE mice treated with vehicle as represented by a significantly lower cumulative clinical score from dosing onset to the end of the study (DPI 16–44; Fig. 1A). However, clinical scoring suffers from generating only ordinal level data and rater bias [20]. To overcome these limitations of clinical scoring, kinematic gait analysis in the sagittal plane was also employed as a sensitive measure of motor deficit in EAE mice. Following a baseline recording of gait prior to disease induction, weekly recordings were taken beginning at DPI 09. Gait parameters were similar for the vehicle and IRX4204 groups from DPI − 02–16. In line with our previous findings, average knee angle decreased from DPI − 02–16, while knee RMS and ankle RMS increased from DPI 09–16 (Fig. 1F, G, H, I) [22]. Each of these deficits were partially reversed following administration of IRX4204. EAE mice that received vehicle maintained a significantly reduced average knee angle and significantly increased knee and ankle RMS values compared to EAE mice that received IRX4204 (Fig. 1F, G, H, I).

### Oral treatment with IRX4204 beginning at peak disease reduces white matter loss and axonal transection in EAE

**Fig. 2:**
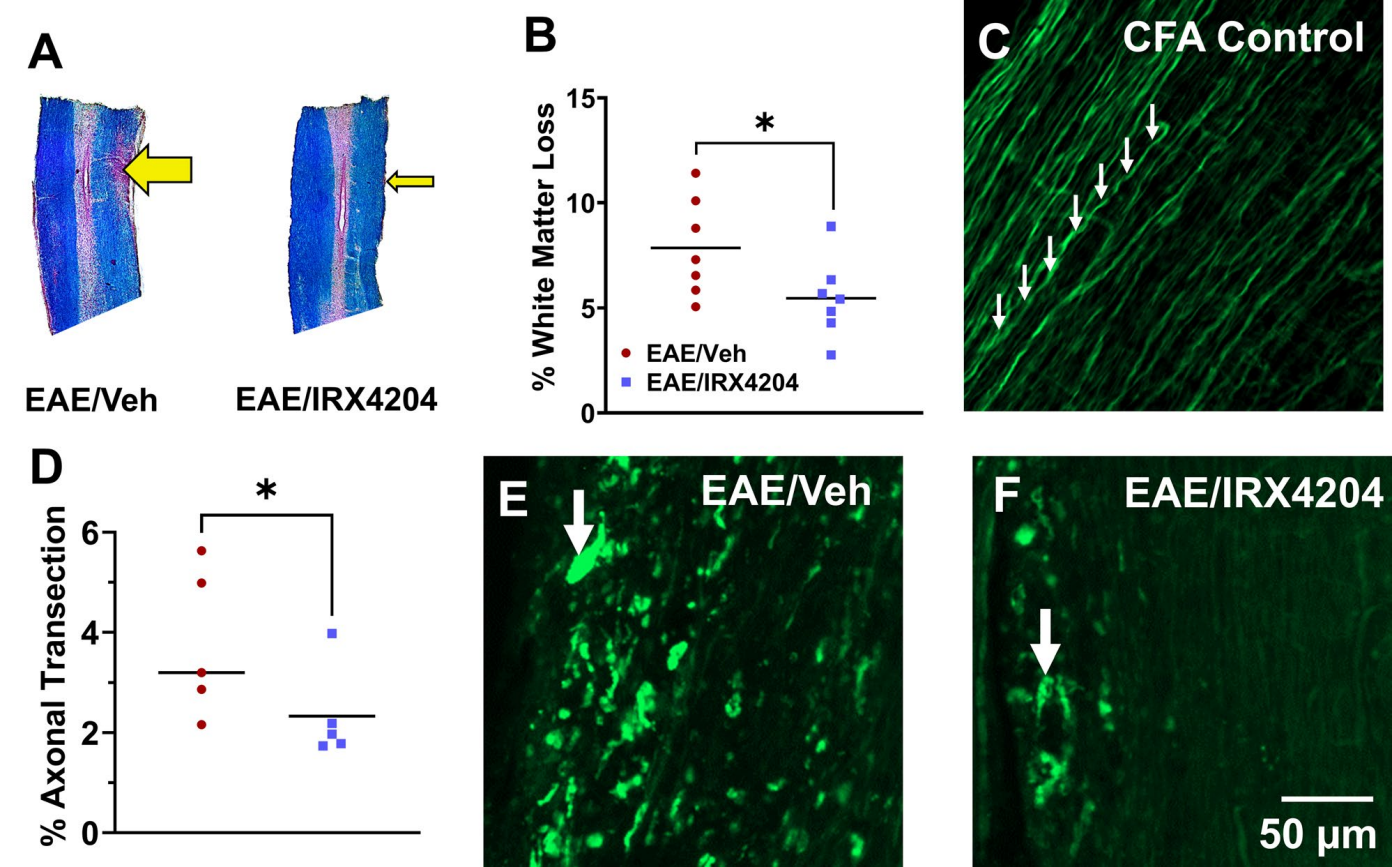
Oral administration of IRX4204 beginning at peak disease (DPI 16) reduces white matter loss and axonal transection in the lumbar spinal cord of EAE mice. (**A**) Representative spinal cord sections from EAE mice treated orally with vehicle (Veh; 5 mL/kg/day; left) or IRX4204 (12 mg/kg/day; right) stained with Eriochrome Cyanine and counterstained with Neutral Red. Arrows depict regions of white matter loss. (**B**) Quantification of regions of white matter loss revealed a significant reduction in white matter loss at DPI 44 in IRX4204/EAE compared to EAE/Veh mice (*n* = 7). (**C**, **E**, **F**) Representative images of eYFP fluorescence in corticospinal axons in white matter regions of spinal cord sections from EAE mice treated orally with Veh (5 mL/kg/day) or IRX4204 (12 mg/kg/day). (**D**) Axon damage, quantified at DPI 44 by calculating percent area of suprathreshold punctate eYFP labelling, revealed a reduction in axonal transection in EAE/IRX4204 relative to EAE/Veh mice (*n* = 5, one-tailed Mann-Whitney *U* test, mean; *p < 0.05)

To assess the effects of IRX4204 on pathophysiological hallmarks of EAE, we measured levels of white matter loss and axonal transection in the spinal cord. EC staining with NR counterstaining were used to quantify white matter loss at DPI 44 (Fig. 2A). Relative to the spinal cords of EAE/Veh mice, EAE/IRX4204 mice showed significantly reduced white matter loss in the lumbar spinal cord by DPI 44 (Fig. 2B). To determine whether this reduction in white matter loss may also be associated with reduced axonal injury, we quantified punctate eYFP labelling in corticospinal axons of EAE Thy1-eYFP mice receiving either vehicle (5 mL/kg/day, p.o.) or IRX4204 (12 mg/kg/day, p.o.) beginning at peak disease (DPI 16; Fig. 2E and F). Quantification of punctate eYFP labelling revealed that EAE/IRX4204 mice showed significantly reduced axonal transection in the lumbar spinal cord compared to EAE mice treated with vehicle by DPI 44 (Fig. 2D).

### Oral treatment with IRX4204 beginning at peak disease increases pro-myelination and reduces pro-inflammatory gene expression in spinal cords of EAE mice

**Fig. 3:**
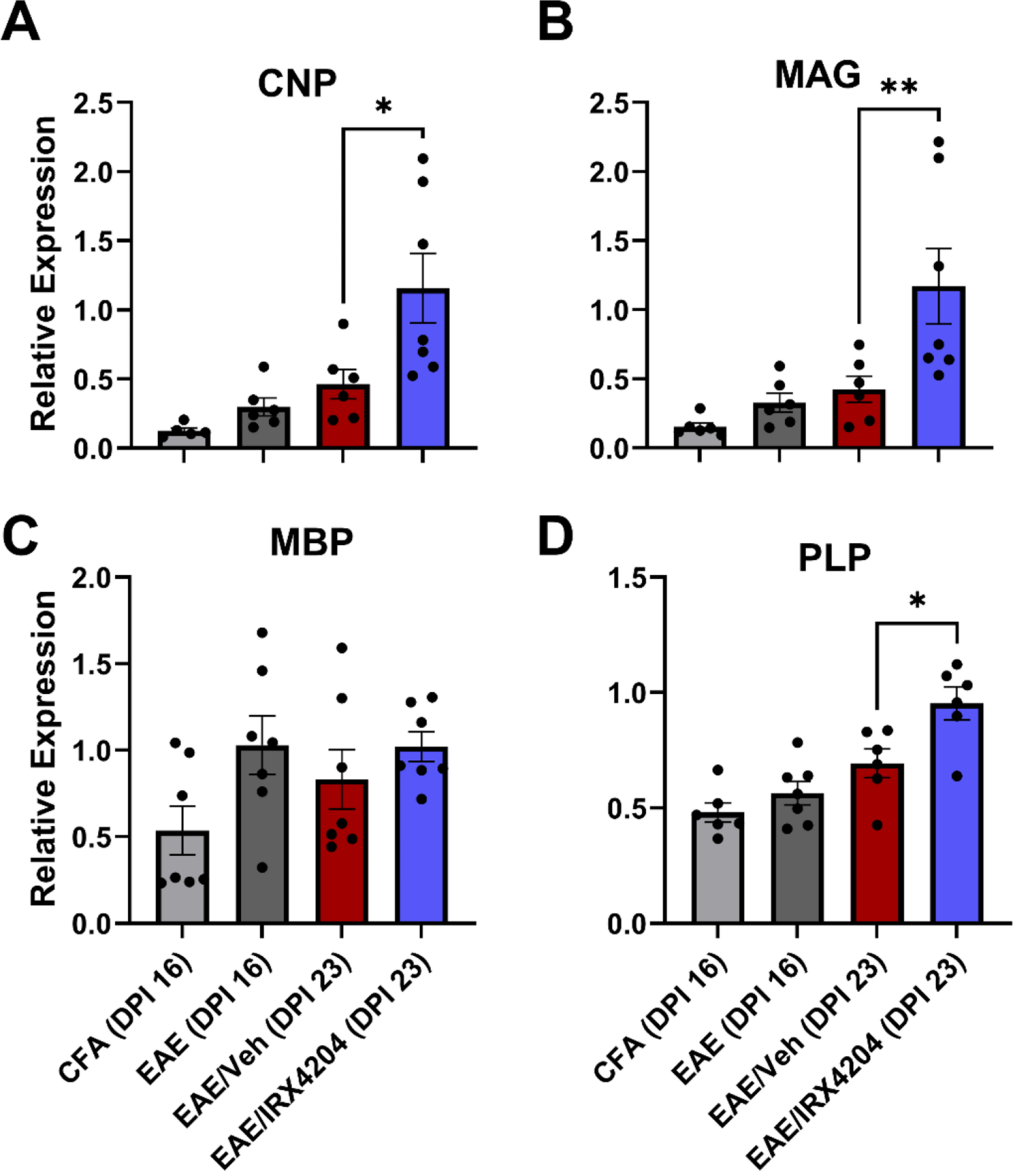
Oral administration with IRX4204 at peak disease (DPI 16) increases pro-myelinating gene expression in the spinal cord of EAE mice. (**A**–**D**) Expression of 2’,3’-Cyclic nucleotide 3’-phosphodiesterase (CNP; **A**), myelin-associated glycoprotein (MAG; **B**), myelin basic protein (MBP; **C**), and proteolipid protein (PLP; **D**) normalized relative to reference gene expression in mouse spinal cord. CNP, MAG, and PLP, expression increased in EAE mice treated with IRX4204 (12 mg/kg/day) compared to EAE mice treated with Veh (5 mL/kg/day) beginning at peak disease (*n* = 7, one-tailed Mann-Whitney *U* test, mean ± SEM; *p < 0.05, **p < 0.01)

We next examined whether reductions in motor deficits, white matter loss, and axonal transection following treatment with IRX4204 were accompanied by increased myelin-associated gene and reduced pro-inflammatory gene expression. For myelin-associated genes, we compared mRNA levels for 2’,3’-Cyclic nucleotide 3’-phosphodiesterase (*CNP*), myelin associated glycoprotein (*MAG*), myelin basic protein (*MBP*), and myelin proteolipid protein (*PLP*) in the spinal cord following one week of treatment with vehicle (5 mL/kg/day, p.o.) or IRX4204 (12 mg/kg/day, p.o.) beginning at peak disease (DPI 16). Relative to vehicle, IRX4204 increased the expression of *CNP*, *MAG*, and *PLP* (Fig. 3A–B, D). By contrast, *MBP* mRNA levels were unchanged.

**Fig. 4:**
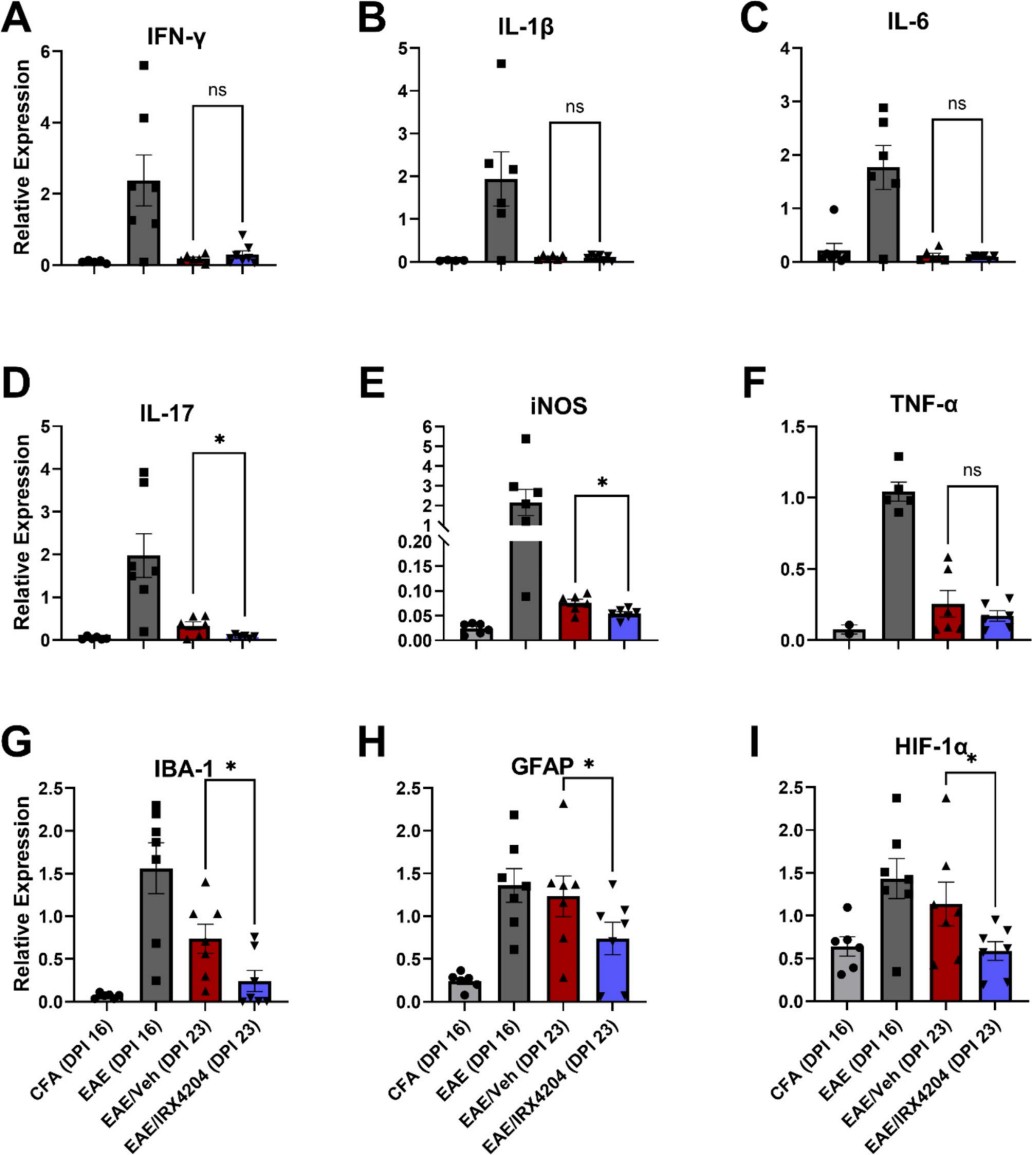
Oral administration with IRX4204 at peak disease (DPI 16) reduces expression of IL-17, iNOS, IBA-1, GFAP, and HIF-1α in the spinal cord of EAE mice. (**A**-**I**) Expression of interferon-γ (IFN-γ; **A**), interleukin 1β (IL-1β; **B**), interleukin 6 (IL-6; **C**), interleukin 17 (IL-17; **D**), inducible nitric oxide synthase (iNOS; **E**), tumour necrosis factor-α (TNF-α; **F**), ionized calcium-binding adapter molecule 1 (IBA-1; **G**), glial fibrillary acidic protein (GFAP; **H**), and hypoxia inducible factor-1α (HIF-1α; **I**) normalized relative to reference gene expression in mouse spinal cord. Expression of IL-17, iNOS, IBA-1, GFAP, and HIF-1α decreased in EAE mice following treatment with IRX4204 (12 mg/kg/day) compared to EAE mice treated with vehicle (5 mL/kg/day) starting at peak disease (*n* = 7, one-tailed Mann-Whitney *U* test, mean ± SEM; *p < 0.05)

We also quantified mRNA levels of the pro-inflammatory genes interferon-γ (*IFN-γ*), interleukin 1β (*IL-1β*), interleukin 6 (*IL-6*), interleukin 17 (*IL-17*), inducible nitric oxide synthase (*iNOS*), tumour necrosis factor-α (*TNF-α*), ionized calcium-binding adapter molecule 1 (*IBA-1*), glial fibrillary acidic protein (*GFAP*), and hypoxia inducible factor-1α (*HIF-1α*) in the spinal cord of these mice. Relative to vehicle, IRX4204 significantly decreased expression of *IL-17*, *iNOS*, *IBA-1*, *GFAP*, and *HIF-1α* in EAE mice (Fig. 4D–E, G–I). No significant differences were observed for expression of *IFN-γ*, *IL-1β*, *IL-6*, and *TNF-α* between both groups (Fig. 4A–C, F).

### IRX4204 promotes remyelination in mice with established EAE

**Fig. 5:**
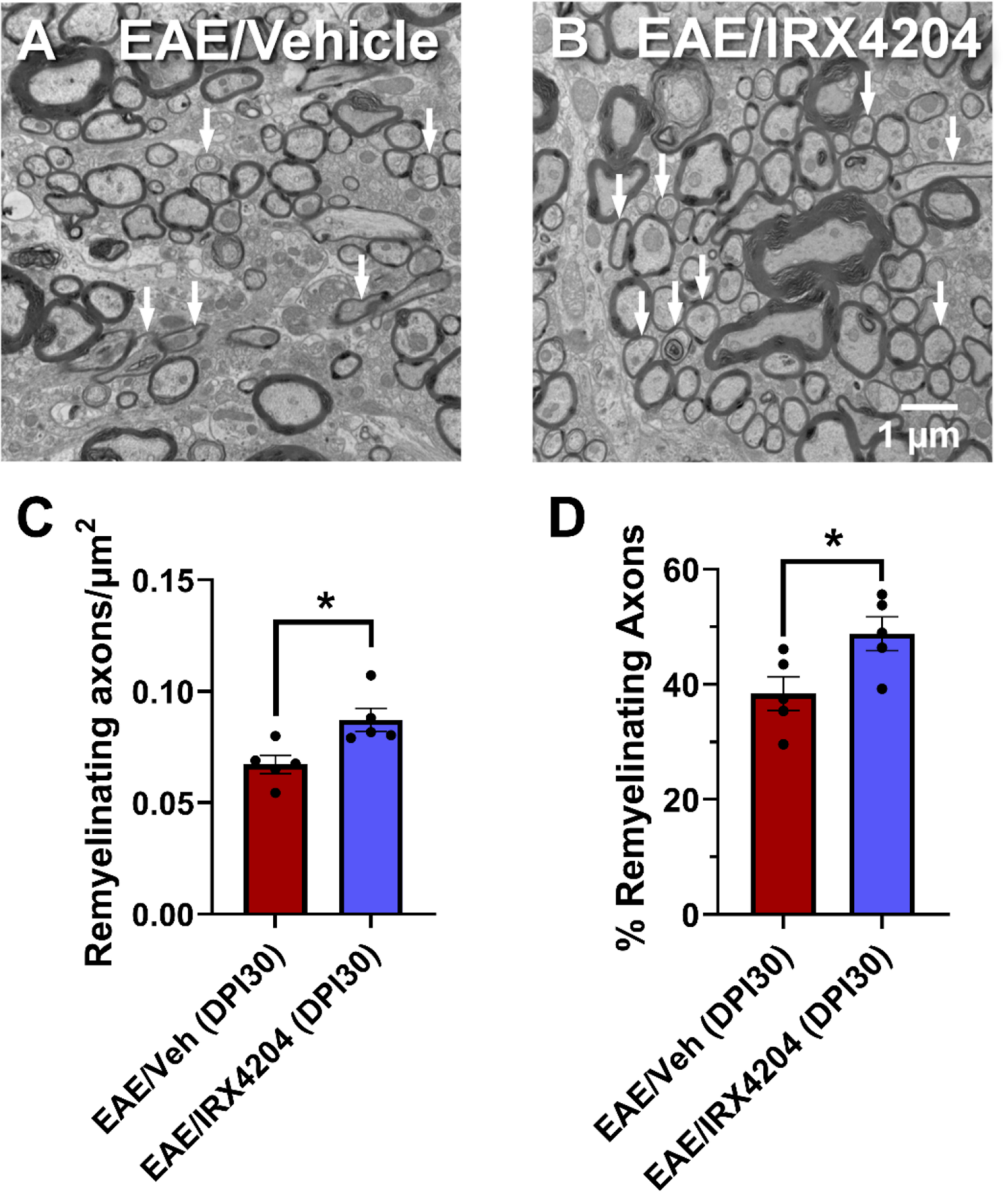
Oral administration with IRX4204 at peak disease (DPI 16) promotes remyelination in the lumbar spinal cord of EAE mice. (**A** and **B**) Representative electron microscopy images of the L3/L4 spinal cord region of EAE mice treated orally with vehicle (5 mL/kg/day; **A**) or IRX4204 (12 mg/kg/day; **B**). Arrows depict remyelinating axons. (**C** and **D**) Axonal counts revealed that remyelinating axons were more abundant (**C**) and made up a higher percentage of axons (**D**) in EAE mice following treatment with IRX4204 compared to EAE mice treated with vehicle starting at peak disease (*n* = 5, one-tailed Mann-Whitney *U* test, mean ± SEM; *p < 0.05)

We next compared remyelination in EAE mice treated with vehicle (5 mL/kg/day, p.o.) or IRX4204 (12 mg/kg/day, p.o.) from DPI 16–30. Coronal imaging of the L3 − 4 region of each spinal cord using transmission electron microscopy (TEM) was performed to identify remyelinating axons (Fig. 5A–B). Remyelinating axons were defined by g-ratios above 0.8 based on prior classification in the EAE mouse model [41]. Axon counts revealed a higher abundance and proportion of remyelinating axons in EAE mice treated with IRX4204 compared to EAE mice treated with vehicle (Fig. 5C–D). However, the g-ratios for vehicle- and IRX4204-treated mice did not differ (Supp Fig. 1).

### IRX4204 attenuates LPS-induced pro-inflammatory gene expression, increases cholesterol efflux gene expression, and elevates phagocytosis in C8-B4 microglial cultures

**Fig. 6:**
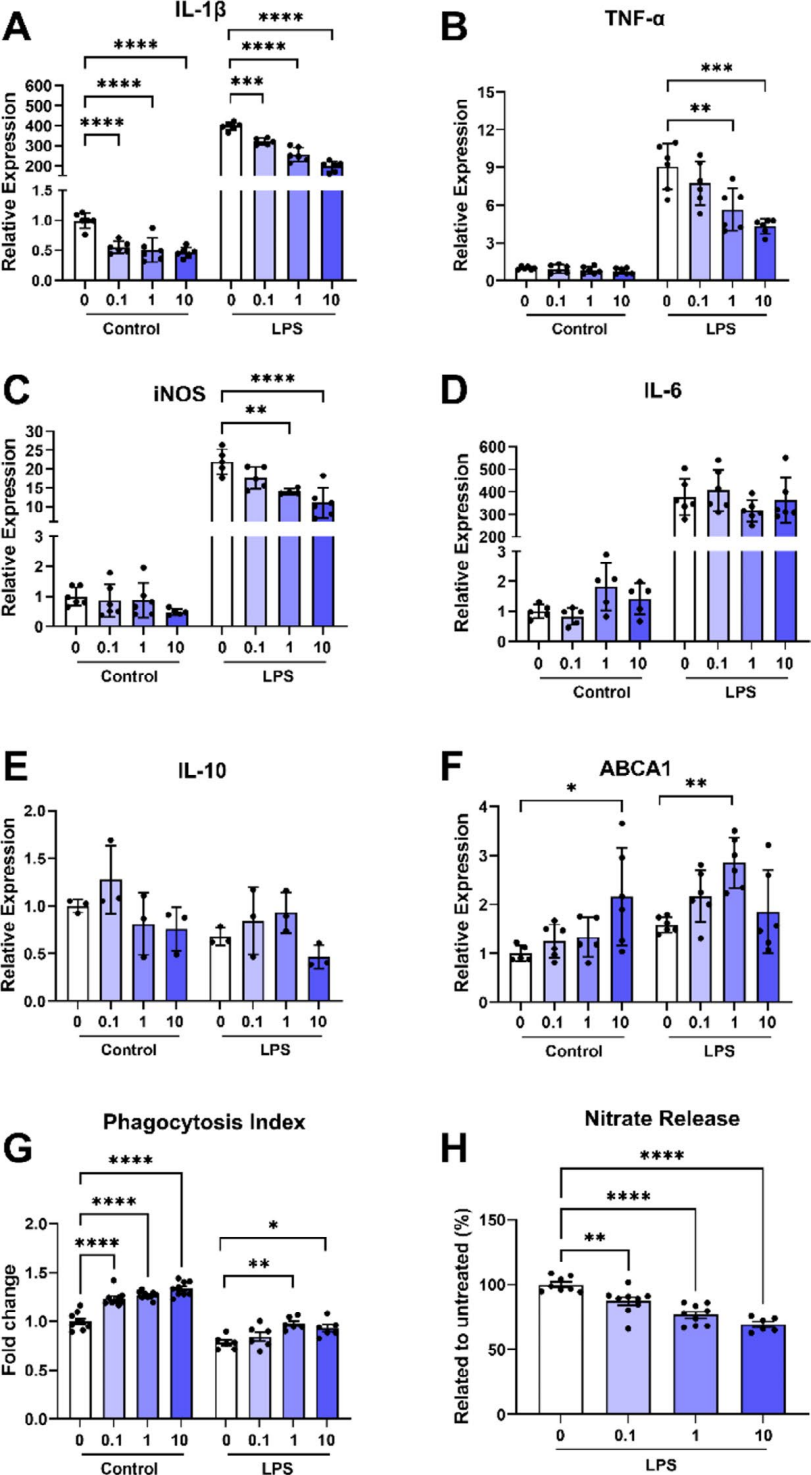
IRX4204 attenuates LPS-induced pro-inflammatory gene expression, increases expression of the cellular transport gene ABCA1, and promotes pro-repair actions of C8-B4 microglia in vitro. (**A**–**F**) IL-1β (**A**), TNF-α (**B**), iNOS (**C**), IL-6 (**D**), IL-10 (**E**), and ABCA1 (**F**), gene expression normalized relative to reference gene expression in microglial cell cultures pre-treated with IRX4204 (24 h) in normal conditions and after treatment with LPS (24 h; *n* = 3–6 wells). Pre-treatment with IRX4204 produced concentration-dependent attenuation of IL-1β, TNF-α, and iNOS expression in microglial cultures incubated with LPS (24 h) and increased ABCA1 expression in microglial cultures at 10 nM in normal conditions and at 1 nM following incubation with LPS. (**G** and **H**) Pre-treatment of these cultures with IRX4204 also increased phagocytic activity in normal conditions at 0.1 nM, 1 nM, and 10 nM (*n* = 6 wells; **G**) and following incubation with LPS at 1 nM and 10 nM. Pre-treatment with IRX4204 at 0.1 nM, 1 nM, and 10 nM was also sufficient to produce a concentration-dependent attenuation of nitrate release (*n* = 6–9 wells; **H**) in microglial cell cultures after incubation with LPS (ordinary one-way ANOVA, Šidák’s multiple comparisons test, *P < 0.05, **P < 0.01, ***P < 0.001, ****P < 0.0001, mean ± SEM)

To assess whether IRX4204 attenuated the expression of pro-inflammatory genes in microglia, we measured gene expression of *IL-1β*, *TNF-α*, *iNOS*, *IL-6*, *CD14*, and *CD206* in C8-B4 microglial cultures pre-treated with PBS, 0.1, 1, or 10 nM of IRX4204. Gene expression was measured for these groups in basal conditions or 24 h following incubation with the potent inflammatory agent LPS. Under basal conditions, microglial cells treated with concentrations of 0.1, 1, or 10 nM IRX4204 showed reduced expression of *IL-1β* compared to microglial cells treated with PBS (Fig. 6A). Compared to cells pre-treated with PBS, cells treated with 0.1, 1, or 10 nM of IRX4204 showed attenuated elevation of *IL-1β* by LPS (Fig. 6A). However, higher concentrations of 1 or 10 nM were required to attenuate LPS-induced increases in *TNF-α* and *iNOS* mRNA levels (Fig. 6B–C). *IL-10* and *ABCA1* mRNA levels were measured to determine the impact of IRX4204 on anti-inflammatory gene expression. Under basal conditions, 10 nM IRX4204 elevated *ABCA1* mRNA levels (Fig. 6F). Under LPS conditions, microglial cells pre-treated with 1 nM of IRX4204 showed increased expression of *ABCA1* compared to microglial cells treated with PBS (Fig. 6F). However, *IL-10* expression was unaltered (Fig. 6E). A crucial component of remyelination involves the activation of pro-repair microglia to clear myelin debris [33, 44]. Cultures pre-treated with IRX4204 showed enhanced pro-repair actions as suggested by increased phagocytic activity (Fig. 6G) and reduced nitrate release (Fig. 6H) following a 24-hr exposure to LPS.

### IRX4204 attenuates LPS-induced pro-inflammatory gene expression in primary astrocyte cultures

**Fig. 7:**
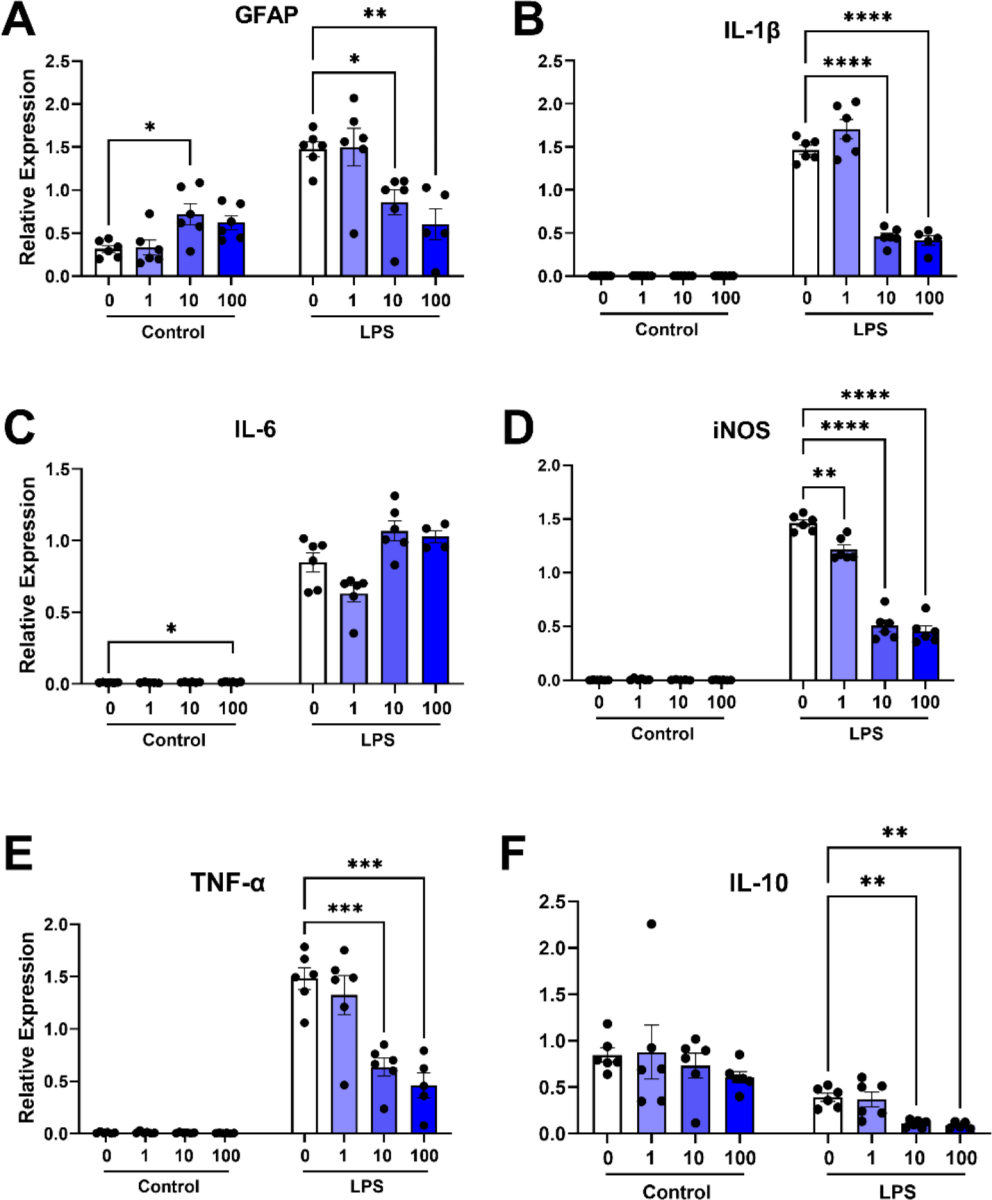
IRX4204 attenuates LPS-induced pro-inflammatory gene expression in primary mouse astrocyte cultures. (**A**–**F**) GFAP (**A**), IL-1β (**B**), IL-6 (**C**), iNOS (**D**), TNF-α (**E**), and IL-10 (**F**), gene expression normalized relative to reference gene expression in astrocyte cultures pre-treated with IRX4204 (24 h) at basal conditions or after incubation with LPS (16 h; *n* = 4–6 wells). IRX4204 produced concentration-dependent attenuation in expression of each of these genes in astrocyte cultures in LPS conditions and increased GFAP expression and IL-6 expression at 10 nM and 100 nM, respectively, in basal conditions (ordinary one-way ANOVA, Šidák’s multiple comparisons test, *P < 0.05, **P < 0.01, ***P < 0.001, ****P < 0.0001, mean ± SEM)

To further evaluate the anti-inflammatory effects of IRX4204 we measured *GFAP*, *IL-1β*, *IL-6*, *iNOS*, *TNF-α*, and *IL-10* mRNA levels in primary astrocyte cultures pre-treated with PBS, 1, 10, or 100 nM of IRX4204. Gene expression was measured for these groups under basal conditions or following incubation with LPS for 24 h. Under basal conditions, *GFAP* mRNA levels were higher in IRX4204 (10 nM) than PBS cultures (Fig. 7A) while astrocytes treated with 100 nM of IRX4204 showed higher expression of *IL-6* (Fig. 7C). In inflammatory conditions, IRX4204 (10 and 100 nM) attenuated the LPS-induced increases in *GFAP* (Fig. 7A), *IL-1β* (Fig. 7B), *iNOS* (Fig. 7D), *TNF-α* (Fig. 7E), and *IL-10* mRNA (Fig. 7F). Furthermore, 1 nM of IRX4204 was sufficient to attenuate LPS-induced *iNOS* expression (Fig. 7D).

### IRX4204 stimulates differentiation in primary rat OPC cultures

**Fig. 8:**
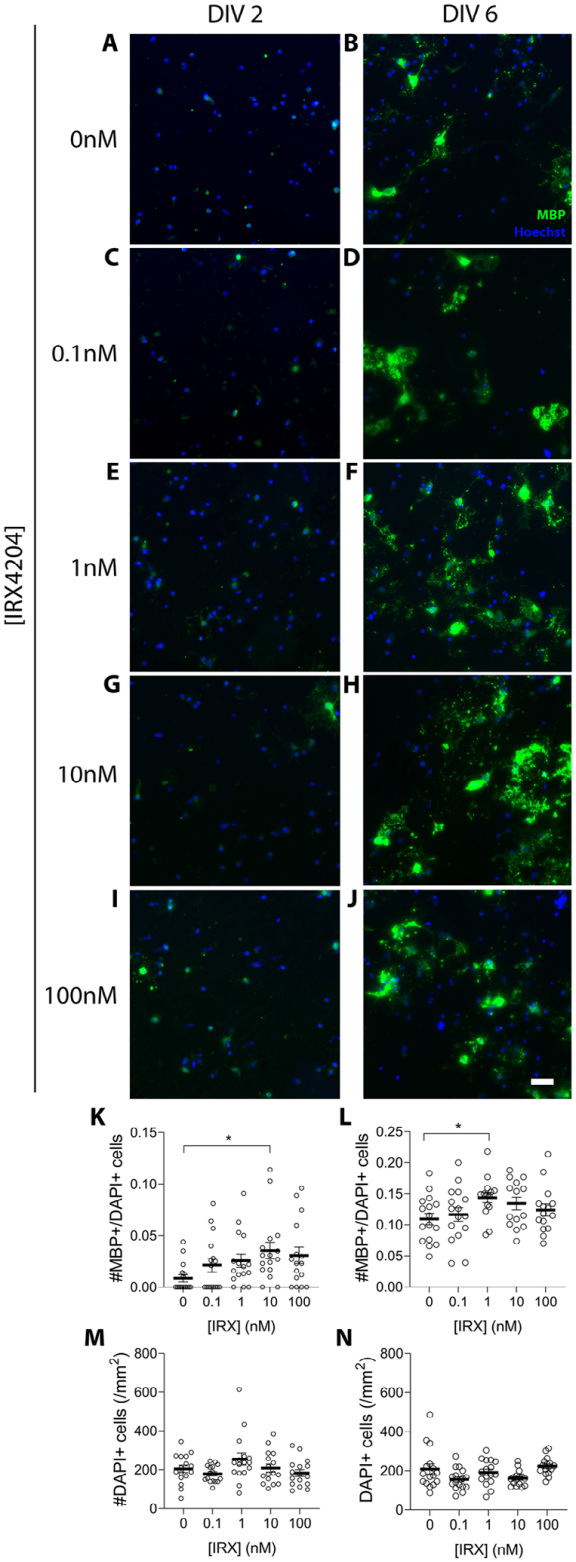
IRX4204 stimulates differentiation of primary rat OPCs in vitro. (**A**–**J**) Representative images of overlain MBP and Hoechst immunofluorescence in OPC cultures. One day following isolation and plating, OPCs were treated with increasing concentrations of IRX4204 for either 24 h (DIV 2; **A**, **C**, **E**, **G**, **I**) or 5 days in vitro (DIV 6; **B**, **D**, **F**, **H**, **J**). (**K**–**N**) Proportion of MBP-positive cells to DAPI-positive cells (**K** and **L**) and number of DAPI-positive cells per mm^2^ (**M** and **N**) after 24 h or 5 days of treatment with increasing concentrations of IRX4204. After 24 h treatment, 10 nM of IRX4204 significantly increased the fraction of MBP immunopositive cells, without altering the total number of cells. After 5 days of treatment, the fraction of MBP immunopositive cells increased with IRX4204 treatment, with a statistically significant increase detected for a concentration of 1 nM IRX4204 (*n* = 14–16 fields/condition; non-parametric Kruskal-Wallis test, Dunn’s multiple comparisons test, *P < 0.05, mean ± SEM; scale bar 50 μm)

To explore the impact of IRX4204 on OPC differentiation, OPCs were isolated from mixed glial cultures and incubated with 0.1, 1, 10, and 100 nM of IRX4204 for either 24 h or 5 days. The fraction of mature oligodendrocytes was assessed by counting cells expressing MBP. After 24 h with IRX4204 (10 nM), the fraction of MBP-immunopositive cells was elevated while the total number of cells remained the same (Fig. 8K and M). Similarly, when OPCs were treated for 5 days, 1 nM of IRX4204 induced an increase in the proportion of mature oligodendrocytes while the overall cell density remained unchanged (Fig. 8L and N).

## Discussion

EAE recapitulates autoimmune-mediated demyelination in MS and has been used successfully to identify immune-based therapeutics that reduce disease relapses. Drug administration in such studies typically occurs before or just after disease onset. However, as is the case for MS, these therapeutics fail to reverse motor deficits and spinal cord injury when administered to mice with established EAE. IRX4204 has been shown to effectively attenuate EAE severity when administered prior to disease onset [16]. Since IRX4204 activates RXRs which are known to promote remyelination [26], we determined whether oral administration of IRX4204 beginning at peak disease improved functional recovery, inflammation, remyelination and axonal integrity in EAE mice. To more accurately assess functional recovery, kinematic gait analysis was employed to obtain highly sensitive and accurate measurements of leg joint movements while mice walked on a treadmill.

Daily EAE clinical scores revealed that treatment with IRX4204 beginning at peak disease enhanced motor recovery as shown by a greater reduction of clinical scores in EAE/IRX4204 compared to EAE/Veh mice that was detected within days of dosing onset. Furthermore, clinical scores for EAE/IRX4204 mice remained lower than EAE/Veh mice until DPI 44 indicating that IRX4204 produced sustained behavioral/motor benefits. These findings were extended by our gait studies which showed that IRX4204 restored leg joint movements in EAE mice. We have previously shown that average knee angle, knee RMS, and ankle RMS are highly sensitive and reliable measures of motor dysfunction and recovery in EAE mice [22, 29]. Not only did IRX4204 largely restore normal average knee angle, knee RMS, and ankle RMS, these improvements persisted until the end of the study. This indicates that IRX4204 produced an enduring improvement of motor function in EAE mice.

IRX4204 administration beginning at peak disease also reduced axonal injury and enhanced remyelination in the spinal cord. Relative to EAE/Veh mice, myelin loss and axonal transection in the spinal cord white matter were less in EAE/IRX4204 mice that were treated once daily with IRX4204 (12 mg/kg) from peak disease (DPI 16) until DPI 44.

To investigate whether reduced white matter loss and axonal transection in the spinal cord were associated with an induction of remyelination, we surveyed expression levels for genes associated with OPC differentiation/myelination in spinal cord tissues of EAE mice following one week of daily vehicle or IRX4204 treatment. In the CNS, *CNP*, *MAG*, *PLP*, and *MBP* are all primarily expressed in oligodendrocytes [45, 55, 59, 69]. CNP promotes the extension of oligodendrocyte processes and maintains the cytoplasmic structure of myelin, MAG regulates oligodendroglial-axonal interactions during formation and maintenance of the myelin sheath, PLP plays a key role in myelin stabilization, and MBP plays a crucial role in myelin compaction [31, 49, 54, 65]. We found that *CNP*, *MAG*, and *PLP* mRNA levels were higher in the spinal cords of EAE/IRX4204 than EAE/Veh mice. No difference was observed between these groups for *MBP* expression, however, *MBP* mRNA are often abundant in MS lesions without being translated into MBP protein [4]. Thus, it is possible that *MBP* mRNA levels may not be reflective of remyelination capacity. Taken together, these findings suggest that IRX4204 promotes the expression of key pro-myelinating genes in EAE.

In addition to increasing the expression of pro-myelinating genes, IRX4204 reduced mRNA levels for pro-inflammatory genes that contribute to chronic inflammation in EAE and in MS. This included IL-17, iNOS, IBA-1, and GFAP. IL-17 is a pro-inflammatory cytokine typically produced by Th-17 cells [23], Th-17 activity and IL-17 production is characteristic of autoimmune disorders such as MS and rheumatoid arthritis [34]. In the present study, we found lower expression of IL-17 in EAE/IRX4204 mice compared to EAE/Veh mice. In line with this finding, earlier studies have shown that targeted inhibition of IL-17 in established EAE enhances recovery [64]. Furthermore, *IBA-1*, a microglial marker of inflammation, and *GFAP*, an astrocytic marker of inflammation, displayed lower levels of expression in EAE/IRX4204 than EAE/Veh mice indicative of reduced microglial and astrocytic reactivity following IRX4204 treatment. Finally, *HIF-1α* expression was also reduced by IRX4204. HIF-1α activity has been shown to sustain inflammation in MS lesions associated with distal dying back oligodendrogliopathy that limits OPC differentiation [36, 67]. Furthermore, hypoxia-like injury is known to be a feature of MS lesions [36]. One study has even shown that inhibition of IL-17 can occur through blockade of HIF-1α activity, ultimately reducing disease severity in established EAE [52]. These findings further suggest that IRX4204 protects the spinal cord of EAE mice by reducing injurious pro-inflammatory events.

To confirm that our transcriptional findings translated to *de facto* remyelination, we quantified remyelinating axons in the spinal cord of EAE/Veh and EAE/IRX4204 mice using TEM. EAE/IRX4204 mice had a greater abundance of remyelinated axons in the lumbar region of spinal cord compared to EAE/Veh mice – confirming that increased pro-myelinating gene expression was associated with remyelination.

A previous study on IRX4204 in EAE mice observed therapeutic benefits for this compound through peripherally-mediated immunomodulation, but did not characterize its direct effects on CNS cells [16]. To elucidate the direct actions IRX4204 has on key CNS cells involved in remyelination, we investigated the effects of IRX4204 in microglial, astrocytic, and OPC cultures. For astrocytes and microglia, we measured levels of gene expression for several cytokines and pro-inflammatory mediators in cultures treated with LPS. Pre-treatment with IRX4204 in microglial cultures led to concentration-dependent attenuation of pro-inflammatory gene expression. This same trend was also observed in astrocyte cultures with IRX4204 attenuating LPS-mediated inflammation in these cells. Furthermore, microglia showed a significant increase in *ABCA1* gene expression at a 1 nM concentration of IRX4204. ABCA1, a cholesterol efflux regulatory protein, plays an important role in microglial support of remyelination. In mice, microglial knockout of *ABCA1* leads to impaired remyelination [6]. These effects likely result from an ABCA1-mediated shift to a pro-repair phenotype which stimulates debris clearance and reduces inflammation [28, 58, 61]. Indeed, we found that treatment of microglia with IRX4204 promoted greater debris clearance by these cells in vitro. Notably, the effective concentrations in our study were up to 100-fold lower than those required for this compound to induce RAR activity – providing new evidence that RXR activation alone is sufficient to enhance debris clearance [60]. Taken together, these experiments suggests that IRX4204 can both promote a shift towards a pro-repair phenotype in microglia and also reduce microglial and astrocyte reactivity to a potent pro-inflammatory stimulus.

In addition to direct effects on microglia and astrocytes, we demonstrated that the RXR agonist IRX4204 enhances OPC differentiation in vitro. Following treatment with IRX4204, OPC cultures exhibited an increased fraction of mature oligodendrocytes without affecting cell density. These findings are consistent with previous studies reporting that IRX4204 and other RXR agonists promote remyelination, without impacting cell proliferation or survival [1, 26, 50]. Following 24 h of treatment a dose-dependent effect is detected between 0.1 and 10 nM, with an increase in the mean number of MBP immunopositive cells at both concentrations, but with the difference only being statistically significant at 10 nM. This is likely due to the 24-hr treatment reflecting an acute effect of the drug, with the higher concentration producing a significant difference. After 5 days of treatment, likely reflecting a steady-state response, prolonged treatment with 1 nM became sufficient to significantly increase the number of MBP immunopositive cells. Although not reaching statistical significance, 5 days of treatment with the 10 nM concentration did also increase the mean number of MBP immunopositive cells. Ultimately, these findings show that IRX4204 can promote OPC differentiation independent from, and in addition to, its effects on inflammation and debris clearance.

Myelin restoration has long been viewed as a primary strategy for neuroprotection in the context of MS. The current study shows that RXR activation by IRX4204 promotes remyelination and axonal protection in vivo in the context of autoimmune-mediated demyelination. Furthermore, IRX4204 led to a recovery of motor function even after deficits were established. The findings from our cell studies add to the growing literature showing that RXR agonists reduce inflammation, initiate glial polarization to a pro-repair phenotype, enhance microglial debris clearance, and promote OPC differentiation [17, 26, 43, 50, 63]. Ultimately highlighting the pleiotropic mechanisms of RXR activation for enhancing remyelination in the context of an inflammatory demyelinating disease. Our current study expands upon previous findings that the RXR serves as a highly promising target for remyelinating therapies in the context of MS [10, 26].

### Study limitations

Although our in vitro studies suggest that IRX4204 may promote myelin repair by acting on multiple glial cell subtypes, we have not conclusively demonstrated that this is the case. IRX4204 has previously been shown to enhance the differentiation of CD4 positive T cells into inducible regulatory T cells and suppress the development of T helper 17 cells in vitro [16]. Hence, it is possible that IRX4204 may also enhance functional recovery by actions on pro-repair T cells. We have calculated the g-ratios for vehicle- and IRX4204-treated EAE mice and found no difference between these groups (Supp Fig. 1). Although axonal conduction along the optic nerve is reduced in EAE mice, the average g-ratios for the optic nerve of healthy and EAE mice have been reported to be similar [24]. This may suggest that the g-ratio does not have sufficient sensitivity to detect small changes in myelin thickness. Indeed, we observed only a small (20%), but significant, difference in remyelinating axons between vehicle- and IRX4204-treated mice. In future studies, advanced imaging methods such as optical coherence tomography or magnetic resonance imaging may complement TEM imaging of myelin by providing further confirmation of the extent of myelin loss and regeneration. While our in vitro and in vivo findings indicate that IRX4204 reduces inflammation, promotes remyelination, and protects axons, future studies are required to confirm that functional recovery resulted from these mechanisms.

## Conclusions

Our findings provide compelling evidence that support the therapeutic potential for IRX4204 as a remyelinating and neurorestorative treatment for MS. Furthermore, the enhanced specificity of IRX4204 for RXRs may address some of the safety concerns that have limited the use of potent RXR agonists clinically. For example, a phase 2a clinical trial with BXT showed modest efficacy for improving secondary outcomes in MS although this compound was not well tolerated in patients [10]. Additionally, data from a single phase I clinical trial with IRX4204 showed that this compound appears to be better tolerated compared to an MS clinical trial with BXT [10, 51]. Taken together with the results of the present study, these clinical findings position IRX4204 as a promising drug to promote functional recovery in patients with progressive forms of MS and inform drug discovery efforts to develop restorative therapeutics for MS by showing that RXR agonism alone is sufficient for effective myelin repair. Future studies directly comparing the efficacy and safety of IRX4204 with other putative remyelinating agents such as bexarotene or clemastine could provide key insights into the most promising drug targets for promoting remyelination in individuals with MS. Furthermore, use of other models of demyelination and remyelination could further distinguish the remyelinating properties of these drugs from their immunomodulatory properties.

## Electronic supplementary material

Below is the link to the electronic supplementary material.

Supplementary Material 1

Supplementary Material 2

## Data Availability

The data from the current study are available from the corresponding author upon reasonable request.
