# Supplementary figures and images for: Selective retinoid X receptor agonism promotes functional recovery and myelin repair in experimental autoimmune encephalomyelitis
Source: Acta Neuropathol Commun. 2024 Dec 21;12:197. doi: 10.1186/s40478-024-01904-x (PMC11662761; doi:10.1186/s40478-024-01904-x)

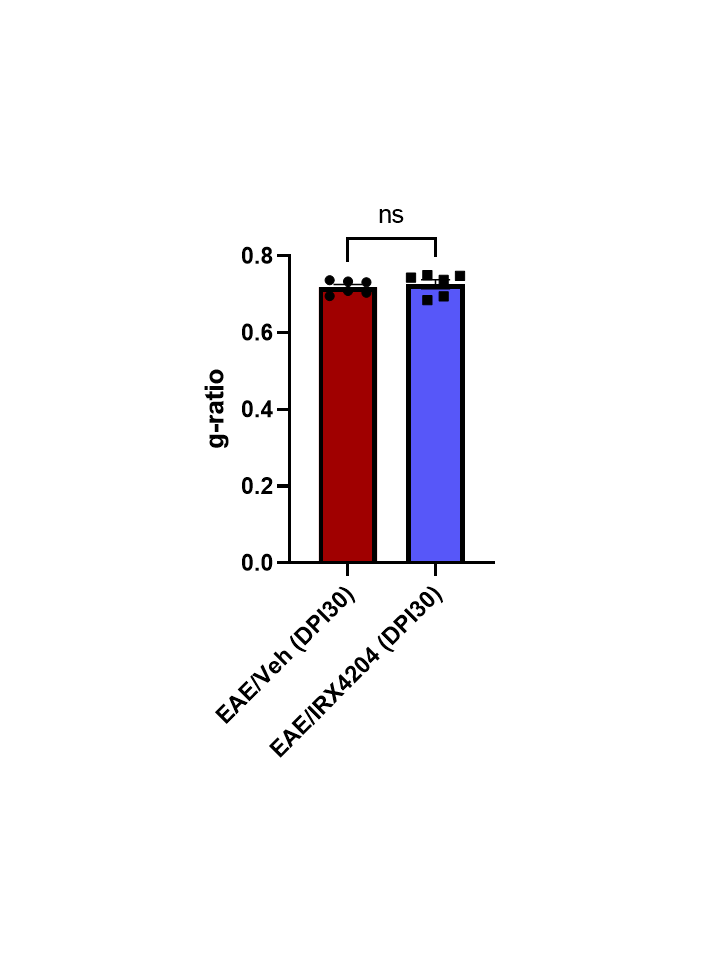

Supplement: Supplementary file 1 — Supplementary Material 1 [file 40478_2024_1904_MOESM1_ESM.tif]
