# Supplementary material for: Selective retinoid X receptor agonism promotes functional recovery and myelin repair in experimental autoimmune encephalomyelitis
Source: Acta Neuropathol Commun. 2024 Dec 21;12:197. doi: 10.1186/s40478-024-01904-x (PMC11662761; doi:10.1186/s40478-024-01904-x)
Supplement: Supplementary file 2 — Supplementary Material 2 [file 40478_2024_1904_MOESM2_ESM.docx]

**Table 1 Primer sequences used for RT-qPCR**

| **Primer** | **Sequence** |
| --- | --- |
| ABCA1 (F) | AAAACCGCAGACATCCTTCAG |
| ABCA1 (R) | CATACCGAAACTCGTTCACCC |
| CNP (F) | TACTTCGGCTGGTTCCTGAC |
| CNP (R) | GCCTTCCCGTAGTCACAAAA |
| GFAP (F) | CGGAGACGCATCACCTCTG |
| GFAP (R) | AGGGAGTGGAGGAGTCATTCG |
| HIF-1a (F) | ACCTTCATCGGAAACTCCAAAG |
| HIF-1a (R) | CTGTTAGGCTGGGAAAAGTTAGG |
| IBA-1 (F) | ATCAACAAGCAATTCCTCGATGA |
| IBA-1 (R) | CAGCATTCGCTTCAAGGACATA |
| IFN-g (F) | ATGAACGCTACACACTGCATC |
| IFN-g (R) | CCATCCTTTTGCCAGTTCCTC |
| IL-10 (F) | GCTCTTACTGACTGGCATGAG |
| IL-10 (R) | CGCAGCTCTAGGAGCATGTG |
| IL-17 (F) | GCTCCAGAAGGCCCTCAGA |
| IL-17 (R) | CTTTCCCTCCGCATTGACA |
| IL-1b (F) | AAATGCCACCTTTTGACAGTG |
| IL-1b (R) | CTGGATGCTCTCATCAGGACA |
| IL-6 (F) | CTGCAAGAGACTTCCATCCAG |
| IL-6 (R) | AGTGGTATAGACAGGTCTGTTGG |
| iNOS (F) | GGCCAGCCTGTGAGACCTTT |
| iNOS (R) | TTGGAAGTGAAGCGTTTCG |
| MAG (F) | CTGCCGCTGTTTTGGATAATGA |
| MAG (R) | CATCGGGGAAGTCGAAACGG |
| MBP (F) | GGCCAGTAAGGATGGAGAGAT |
| MBP (R) | CCTCTGAGGCCGTCTGAGA |
| PLP (F) | CCAGAATGTATGGTGTTCTC |
| PLP (R) | GGCCCATGAGTTTAAGGAC |
| TNF-a (F) | CAGGCGGTGCCTATGTCTC |
| TNF-a (R) | CGATCACCCCGAAGTTCAGTAG |
| GAPDH (F) | AGGTCGGTGTGAACGGATTTG |
| GAPDH (R) | GGGGTCGTTGATGGCAACA |
| HPRT1 (F) | TCAGTCAACGGGGGACATAAA |
| HPRT1 (R) | GGGGCTGTACTGCTTAACCAG |

^a^Forward primer denoted by (F).

^b^Reverse primer denoted by (R).
